# Livestock shifts lepidopteran herbivore community due to intraguild elimination in Mediterranean agroforestry systems

**DOI:** 10.1002/eap.3088

**Published:** 2025-01-11

**Authors:** Álvaro Gaytán, Tara Canelo, Carlos Pérez‐Izquierdo, Raúl Bonal

**Affiliations:** ^1^ Institute of Natural Resources and Agrobiology of Seville (IRNAS‐CSIC) Seville Spain; ^2^ Departamento de Ingeniería del Medio Agronómico y Forestal, Grupo de Investigación Forestal, INDEHESA Universidad de Extremadura Plasencia Spain; ^3^ Centro de Ecologia Aplicada “Prof. Baeta Neves” (CEABN), Instituto Superior de Agronomia Universidade de Lisboa Lisboa Portugal; ^4^ Department of Biodiversity, Ecology and Evolution, Faculty of Biological Sciences Complutense University of Madrid Madrid Spain

**Keywords:** agroforestry systems, community composition, insect defoliation, livestock, *Quercus ilex*

## Abstract

Livestock grazing and trampling have been shown to reduce arthropod populations. Among arthropods, defoliating lepidopterans are particularly important for their impact on trees, the keystone structures of agroforestry systems. This study investigates the impact of livestock on the community of defoliating lepidopterans in agroforestry systems. We conducted both experimental and observational studies in the mid‐west and western regions of the Iberian Peninsula. In our field experiment, we sampled lepidopteran caterpillars in areas with livestock and in areas where livestock had been excluded for short and long periods. To validate our experimental results, we conducted a field survey across seven areas with and without livestock in the western Iberian Peninsula. Our findings revealed that after 2 years from the start of the experiment, the exclusion of livestock led to an increase in the abundance and species richness of lepidopterans, as well as shifts in their community composition. Our experimental findings were corroborated by our field survey. Interestingly, we found that the differences in community composition among exclusions were primarily due to species nestedness. Livestock exclusion consistently favored species that pupate in the ground, suggesting that livestock can alter the lepidopteran community by predating or accidentally trampling these species. This study underscores the significant role livestock play in modifying the community of defoliating lepidopterans in Mediterranean agroforestry systems (oak savannahs), with important implications for food webs and species interactions.

## INTRODUCTION

Agroforestry systems are productive systems that predominantly combine forestry and agricultural management with free‐range livestock (Moreno & Pulido, 2009). Livestock significantly influences terrestrial ecosystems by modifying vegetation structures and altering the physical and chemical properties of soil and plants (Asner et al., 2004; Bakker et al., 2004; Peco et al., 2006). Furthermore, these effects of livestock can profoundly impact other animal populations, such as insects. Studies have shown that livestock grazing and movement can lead to a reduction in arthropod populations by accidentally eating or trampling them (Bonal & Muñoz, 2007; Canelo et al., 2021; Gómez & González‐Megías, 2002). A large community of arthropods depends on trees (Gaytán et al., 2021; Manning et al., 2006), which are often identified as keystone structures in agroforestry systems also for livestock since they consume their fruits and take advantage of their shade (Moreno & Pulido, 2009; Rodriguez‐Estevez et al., 2012). The exploration of interactions between livestock and arthropods in agroforestry systems is particularly intriguing. It could potentially aid in regulating populations of certain pests, such as defoliating insects or cause changes in their community structure (Canelo et al., 2021; Gish et al., 2011; Valburg, 1992). Nonetheless, intensive livestock management is threatening natural tree recruitment and conservation (López‐Sánchez et al., 2016).

Evergreen oaks (*Quercus* spp.) play a pivotal role in both agroforestry systems and natural forests of southern Europe. In agroforestry systems, trees are a primary shade source for free‐range livestock and their fruits (acorns) contribute significantly to the production of high‐quality meat (Rodriguez‐Estevez et al., 2012). These evergreen oaks (mainly *Quercus ilex* and *Quercus suber* in Iberian oak savannahs) are primarily defoliated by lepidopterans belonging to geometridae, noctuidae, and tortricidae families, where the most abundant species are *Archips xylosteana*, *Dryobotodes* spp., *Catocala nymphagoga*, or *Tortrix viridana* (Gaytán et al., 2018). These defoliating lepidopterans reach their peak feeding period during spring (López‐Sánchez et al., 2016; Robinson et al., 2010). This defoliation often results in a substantial loss of leaf mass in Mediterranean forests (Pollastrini et al., 2019), and such losses can lead to a significant reduction in tree growth and reproduction (Canelo et al., 2018; Zvereva & Kozlov, 2014). Interestingly, livestock can have both positive and negative effects on the community of defoliating insects. On one hand, the presence of livestock can promote insect defoliation by increasing the palatability (nitrogen availability) of plants, thereby enhancing the quality of leaves (Erelli et al., 1998; Leghari et al., 2016; Tripler et al., 2002). On the other hand, free‐range livestock consume plants that serve as food and habitat for insects. Their coexistence in the same landscape leads to multitrophic interactions, such as intraguild elimination or inadvertent insect consumption by large ungulates (Canelo et al., 2021; Gish et al., 2011; Losey & Denno, 1998; Suominen et al., 1999; Suominen & Olofsson, 2000; Valburg, 1992; Zamora et al., 1993). However, the impact of livestock on the community of defoliating lepidopterans in agroforestry systems remains largely unexplored.

Besides being killed by their natural enemies, lepidopterans may also be eaten by other animals at the same trophic level (intraguild elimination). For instance, large ungulates can significantly impact insect populations that feed on seeds. This is because they feed on acorns during the period when the larvae inhabit these fruits (Bonal & Muñoz, 2007; Canelo et al., 2021; Valburg, 1992). Similarly, large ungulates may consume or trample other herbivorous insects while grazing or browsing as aphids or gall inducing insects (Gish et al., 2011; Losey & Denno, 1998; Rambo & Faeth, 1999; Zamora et al., 1993), but they have not a particularly vulnerable stage in their life cycle as tree defoliating lepidopterans do. Defoliating lepidopterans are inaccessible to large herbivores during their larval stage, but this is not always the case during the pupal stage. Depending on the species, the pupal stage can occur on branches, the trunk, or on the ground (either on the surface or slightly buried in the litter), and its duration varies from a few weeks to several months, depending on the life history of each species (Gaytán et al., 2018). Species that undergo their pupal stage in the ground over extended periods, such as many geometrid and noctuid species (Jonko, 2023), could be more vulnerable to livestock. This is because the risk of predation or accidental trampling by livestock and wild mammals is higher than for those species that pupate on branches. Therefore, we anticipate that areas with livestock might be dominated by species that avoid pupating on the ground.

Our primary objective was to determine the extent to which livestock influences the defoliating lepidopteran community within Mediterranean agroforestry systems. We also aimed to specifically identify which species are favored or disfavored by the presence of livestock, given the importance of certain species or groups of species within the forest history of southern Europe as *T. viridana* (Soria & Notario, 1990) or *Lymantria dispar* (Bernal et al., 2023). To achieve this, we employed both observational and experimental methodologies to evaluate the impact of livestock on this community. We capitalized on a field experiment where we sampled caterpillars at three areas. These areas were dominated by the most prevalent tree species within southern European agroforestry systems, namely, the Mediterranean oak (*Q. ilex* L.). At each site, we sampled eight trees in areas that had been devoid of livestock for over 15 years, eight trees within short‐term exclusions (2 years), and eight trees in areas with livestock. To gauge the generalizability of our experimental results, we conducted a field survey of caterpillars at seven areas (four with livestock and three without livestock) in western Iberian Peninsula. Our investigation was guided by the following specific questions:

1. Do the abundance of lepidopterans, species richness, evenness, and community composition of the herbivore community change with the presence of livestock?
2. Are lepidopteran species that pupate in the ground more abundant in areas without livestock?
3. Are differences in the herbivore community composition among areas with and without livestock a product of species turnover or nestedness?

We expect that livestock cause profound changes in the community of defoliating insects. More specifically, we expect that those herbivore species that usually pupate in the ground would be less abundant in those areas with livestock due to intraguild elimination. We think that lepidopteran species pupating in the ground will be substituted by other species that complete most of their life cycle in tree canopies because they would be less vulnerable to livestock trampling or accidental feeding.

## METHODS

### Study system

Oak savannahs (so called “dehesas”) are agroforestry systems composed by interspersed Mediterranean oaks (*Q. ilex* L.) within a grassland matrix, where the main economic activity is the free‐range livestock (mainly cattle, sheep, and pigs; Moreno & Pulido, 2009). The soil in all study areas is acid, with both low nutrient and organic matter content. The study areas are located in the mid‐west of the Iberian Peninsula, where high livestock densities may even lead to the formation of bare soils (see Appendix S1: Table S1 for details on stocking densities the study area). The climate in the study areas is Mediterranean with hot summers with yearly mean temperatures of 16°C, reaching 33°C during July and August, and an annual precipitation of 623.1 mm (Moreno & Pulido, 2009). Mediterranean oaks harbor a large community of defoliating lepidopterans, where the most represented families are geometrids, noctuids, and tortricids (Gaytán et al., 2018; Jonko, 2023). These species differ in their potential susceptibility to the influence of livestock, which is determined by where they pupate (ground or tree canopy) and the duration of the pupal stage. Geometrids usually use silk to descend to the ground for pupation, noctuids pupate on the ground, beneath leaf litter or even in bark roughness, and tortricids are leaf rollers pupating in the tree canopy (Gaytán et al., 2018).

### Field experiment and field survey

**FIGURE 1:**
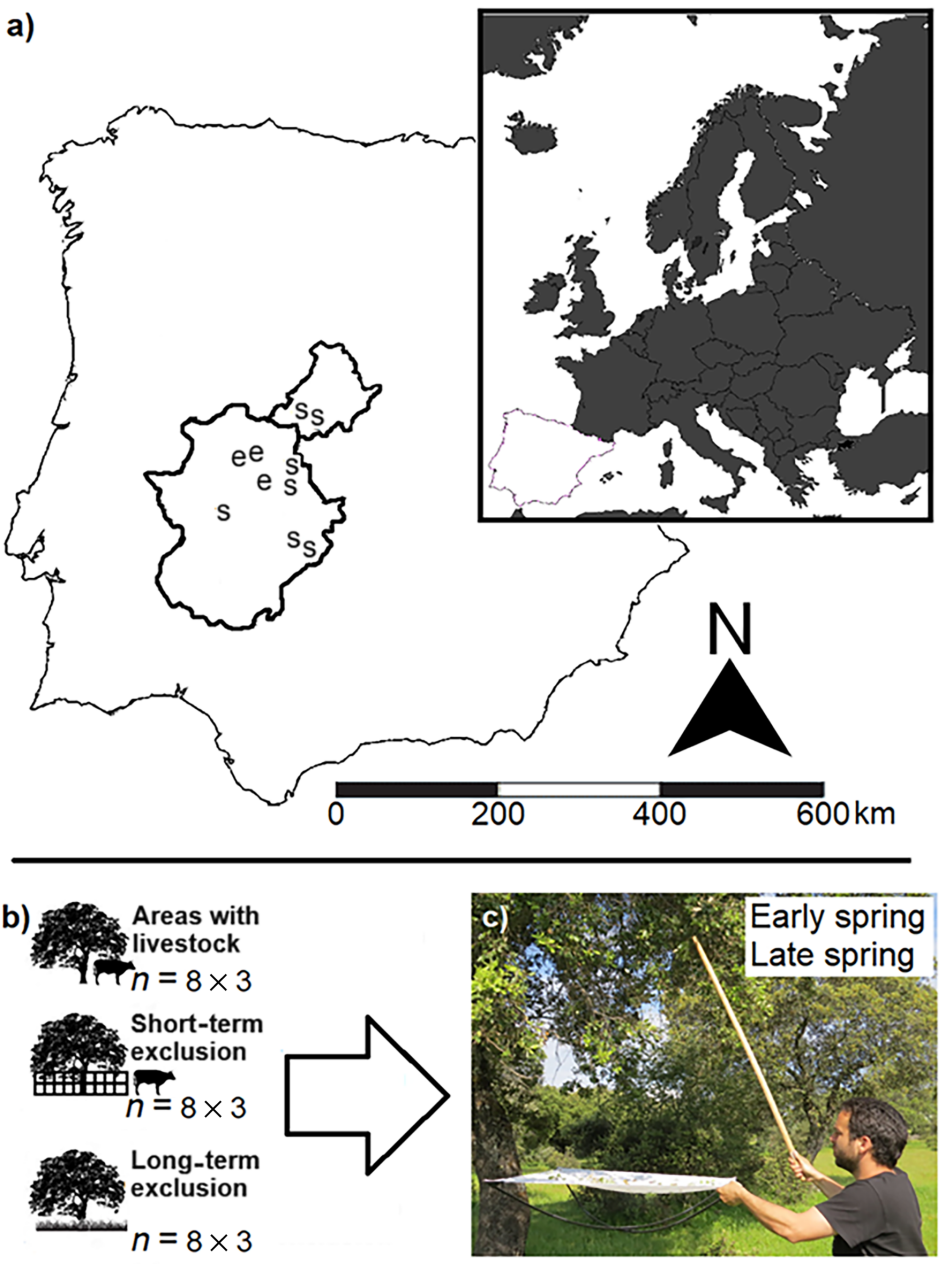
Overview of the experimental design and field sampling: (a) map with the location of the experimental (marked with “e”) and survey areas (marked with “s”); (b) representation of each treatment in the experiment, the number of trees per site (*n* = 8), and the number of areas (*n* = 3); and (c) the sampling approach. Photo credit: Raúl Bonal. Silhouettes: www.phylopic.org.

In April 2016 (early spring), we selected three areas in the mid‐west of the Iberian Peninsula that combined areas with and without livestock (Figure 1). In each site, we (1) selected eight large *Q. ilex* trees ranging from 8 to 15 m in height placed in areas without livestock during >25 years (long‐term exclusion), (2) created short‐term exclusions by fencing eight trees placed in areas with livestock, and (3) selected eight trees sited in areas with livestock (Figure 1) resulting in 72 study trees (8 trees × 3 treatments × 3 areas). In early spring, we sampled each tree by shaking their reachable branches four times per tree and sampling, and we collected the falling caterpillars on a white cloth of a fixed surface (1 × 1 m) placed beneath (Figure 1; cf. Ruiz‐Carbayo et al., 2017). We identified the collected caterpillars at species level based on morphological characters (Gaytán et al., 2018). We repeated the field sampling in May 2016 (late spring), and later in 2017 both in early and late spring (i.e., two times per year), collecting 38 lepidopteran species from 11 families (Table 1). Finally, data from the four sampling periods were pooled for statistical analyses. From April to early May 2019, we surveyed caterpillars in seven areas in the western of the Iberian Peninsula, from which four areas had livestock and three without livestock during the last 10 years using the same methodology (Figure 1). The areas selected for the field survey were distributed in pairs of areas with and without livestock except for one separated site with livestock for which we did not find a site without livestock nearby (Figure 1). In each site, we surveyed 15 trees resulting in 105 trees (15 trees × 7 areas). Livestock type and density of each experimental and survey area are detailed in the Appendix S1: Table S1.

**TABLE 1:** Overview of sampled species with their families, place of pupal stage (ground or tree), and number of individuals collected in each treatment of the experiment.

Species	Family	Pupal stage	Control	Short‐term exclusion	Long‐term exclusion
*Adactylotis gesticularia*	Geometridae	Tree	0	2	0
*Agriopis leucophaearia*	Geometridae	Ground	0	0	3
*Agriopis marginaría*	Geometridae	Ground	2	1	18
*Amphipyra berbera*	Noctuidae	Tree	0	1	0
*Apocheima hispidaria*	Geometridae	Ground	0	1	4
*Archips xylosteana*	Tortricidae	Tree	78	86	23
*Bena bicolorana*	Nolidae	Tree	4	1	5
*Catocala conjuncta*	Erebidae	Tree	0	1	0
*Catocala dilecta*	Erebidae	Tree	0	0	1
*Catocala nymphagoga*	Erebidae	Tree	109	126	162
*Catocala promissa*	Erebidae	Tree	0	0	1
*Colotois pennaria*	Geometridae	Tree	3	0	4
*Dicycla oo*	Noctuidae	Tree	0	2	0
*Dryobota labecula*	Noctuidae	Tree	33	29	71
*Dryobotodes eremita*	Noctuidae	Ground	48	47	64
*Dryobotodes monochroma*	Noctuidae	Ground	43	75	102
*Dryobotodes roboris*	Noctuidae	Ground	1	3	2
*Dryobotodes tenebrosa*	Noctuidae	Ground	7	7	5
*Ennomos quercaria*	Geometridae	Ground	6	3	9
*Erannis defoliaria*	Geometridae	Ground	0	1	4
*Eupithecia cocciferata*	Geometridae	Ground	5	6	23
*Eupithecia irriguata*	Geometridae	Ground	6	12	17
*Eupithecia massiliata*	Geometridae	Ground	8	4	16
*Favonius quercus*	Lycaenidae	Tree	1	2	0
*Harpiya milhauseri*	Notodontidae	Ground	0	2	0
*Lymantria dispar*	Erebidae	Tree	1	0	2
*Malacosoma neustria*	Lasiocampidae	Tree	11	26	22
*Nycteola columbana*	Nolidae	Tree	1	8	5
*Orthosia cerasi*	Noctuidae	Ground	1	1	0
*Orthosia cruda*	Noctuidae	Ground	4	9	8
*Peribatodes ilicaria*	Geometridae	Ground	3	2	0
*Phycita torrenti*	Pyralidae	Tree	73	94	82
*Phyllodesma suberifolia*	Lasiocampidae	Tree	1	0	2
*Satyrium esculi*	Lycaenidae	Tree	0	1	1
*Tortricodes alternella*	Tortricidae	Tree	32	64	184
*Tortrix viridana*	Tortricidae	Tree	173	175	159
*Watsonalla uncinula*	Drepanidae	Ground	2	5	4
*Xanthia ruticilla*	Noctuidae	Ground	1	2	14

### Statistical analysis

To analyze data from our field experiment, we tested if the abundance of lepidopterans (number of individuals) (Equation 1), species richness (Equation 2), evenness (Equation 3), and community composition (Equation 4) differed with the degree of exclusion (LEX). In models with experimental data, the degree of exclusion had three levels (with livestock and short‐ and long‐term livestock exclusion), while in models with observational data from the field survey, it had two levels (with and without livestock exclusion). Since we included species richness and evenness, we considered redundant to include other diversity indices, which depend on species richness and incorporate species evenness (McCune & Grace, 2002). We calculated species richness and Pielou's evenness index using the function *estimate_richness* from the R package *Phyloseq*, which performs standard alpha diversity estimates by operating on the cumulative population of each sample (McMurdie & Holmes, 2013).

We fitted generalized linear mixed models using the function *lmer* in the R package *lme4* (Bates et al., 2015; R Core Team, 2023), specifying a Gaussian distribution with an identity link, and using the function *Anova* in the R package *car* to test for significance (Weisberg, 2019). We inspected the distribution of the residuals of each model to check for normality and heteroscedasticity, and no models required transformation of the response variable. For models on species richness, we added the number of individuals collected in each tree (IND) per sampling as a covariate to account for the potential effect of sampling size on the number of species (Equation 2). In all models, we included the nested random factors sampling area (AREA) and tree identity (TREE) to account for potential differences among areas and for repeated measurements among sampling periods respectively. We used the function *emmeans* from the R package *emmeans* (Lenth, 2020) for paired contrasts among treatments. For the multivariate response variable community composition, we used the function *adonis2* in the R package *vegan* (Oksanen et al., 2015) with Bray–Curtis dissimilarity. Since random factors cannot be specified in *adonis2*, we added the described random factors as fixed factors in the community composition models. We used linear permutational multivariate ANOVA (PERMANOVA) for the multivariate response variable community composition. We used the same set of models with data prior to the establishment of the experiment to check if there were initial differences in the community of lepidopterans among the short‐ and long‐term exclusions. We calculated the marginal *R* ^2^ for the models using the function *r.squaredGLMM* in the R package *MuMIn* (Barton, 2009).

(1) $$Y_{\mathrm{ij}}\sim \beta_{0}+\beta_{1}\;{\mathrm{LEX}}_{\mathrm{ij}}+u_{1}\;{\text{AREA}}_{\mathrm{ij}}+u_{2}\;{\text{TREE}}_{\mathrm{ij}}+\varepsilon$$

(2) $$Y_{\mathrm{ij}}\sim \beta_{0}+\beta_{1}\;{\mathrm{IND}}_{\mathrm{ij}}+\beta_{2}\;{\mathrm{LEX}}_{\mathrm{ij}}+u_{1}\;{\text{AREA}}_{\mathrm{ij}}+u_{2}\;{\text{TREE}}_{\mathrm{ij}}+\varepsilon$$

(3) $$Y_{\mathrm{ij}}\sim \beta_{0}+\beta_{1}\;{\mathrm{LEX}}_{\mathrm{ij}}+u_{1}\;{\text{AREA}}_{\mathrm{ij}}+u_{2}\;{\text{TREE}}_{\mathrm{ij}}+\varepsilon$$

(4) $$Y_{\mathrm{ij}}\sim \beta_{0}+\beta_{1}\;{\mathrm{LEX}}_{\mathrm{ij}}+\beta_{2}\;{\text{AREA}}_{\mathrm{ij}}+\beta_{3}\;{\text{TREE}}_{\mathrm{ij}}$$

We used linear discriminant analysis effect size (LEfSe) algorithms (Segata et al., 2011) to examine which species differed in abundance in areas with and without livestock in the experiment and in the field survey. LEfSe algorithms were used to identify those lepidopteran species that are significantly more abundant in one of our treatments. To test to what extent differences in community composition among treatments and between areas with and without livestock by species turnover or nestedness, we used the R package *betapart* (Baselga & Orme, 2012). We analyzed differences in community composition among treatments and between areas with and without livestock by computing dissimilarity values among treatments in pairwise comparisons, where we partitioned the total β‐diversity (Bray–Curtis dissimilarity) into two indices, where β‐*STU* is the turnover component and β‐*SNE* is the species nestedness component (Culp et al., 2019).

## RESULTS

**FIGURE 2:**
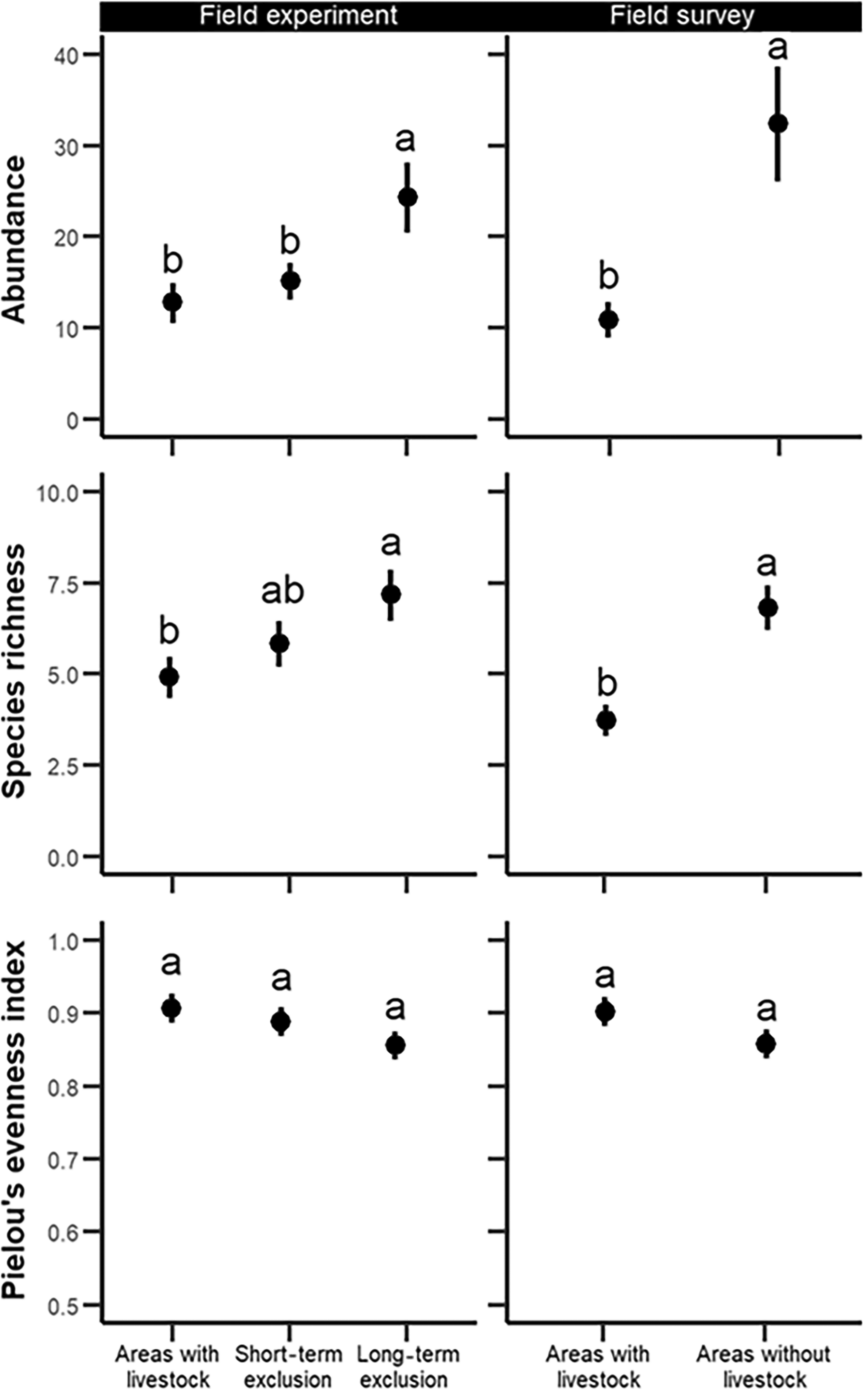
The impact of treatments (field experiment) 2 years after the start of the experiment and the presence of livestock (field survey) on abundance, species richness, and evenness (mean ± SE). Letters indicate significant differences among treatments.

**FIGURE 3:**
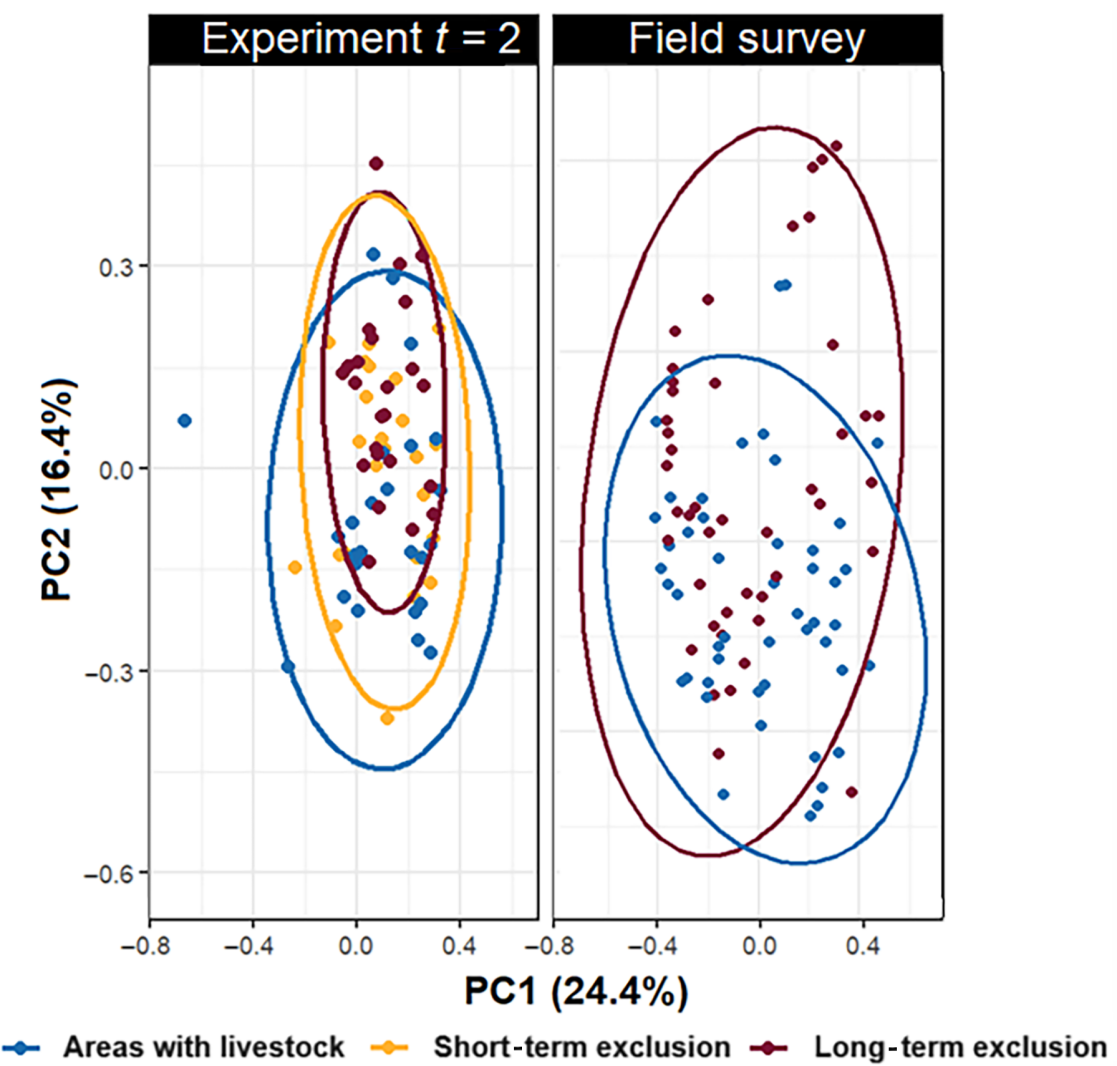
Differences in the community composition of defoliating lepidopterans among treatments (areas with livestock, short‐term exclusion, and long‐term exclusion) 2 years after the start of the experiment and in the field survey. Visualization is based on principal components analysis (PCA) using Bray–Curtis metrics.

**FIGURE 4:**
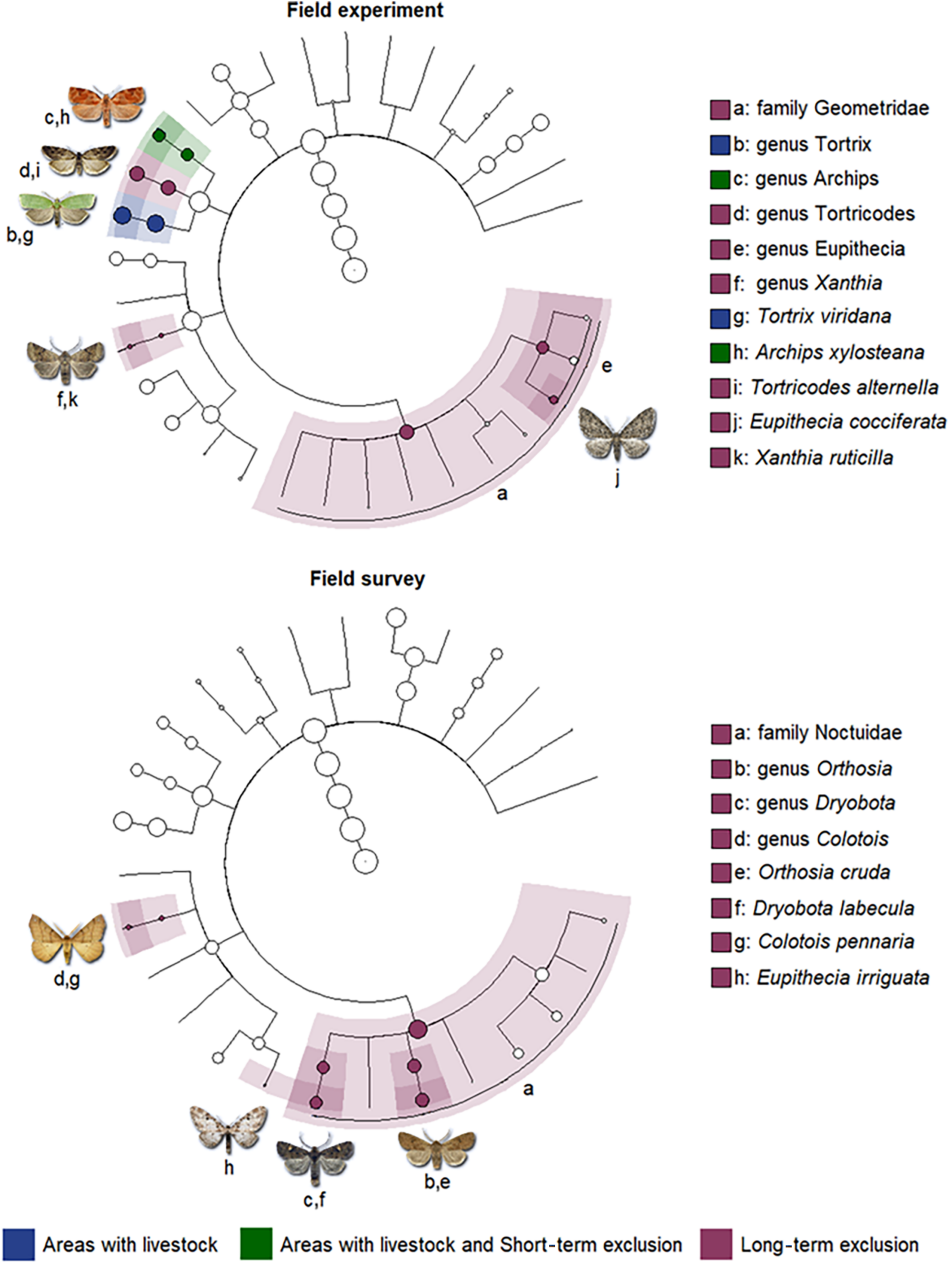
Cladograms of linear discriminant analysis effect size analysis of herbivore abundance from order to species level (*p* < 0.05) for the field experiment and the field survey. The lines represent taxonomic levels, extending from the domain at the center of the cladogram to the species level at the periphery. The colored areas indicate taxonomic groups that are significantly more abundant in different conditions: areas with livestock, areas with livestock and short‐term exclusion, and areas with long‐term exclusion. Depictions highlight species that are significantly more abundant in a specific treatment, labeled with letters, which correspond to the key. Silhouettes: www.lepidoptera.eu.

Overall, long‐term livestock exclusion increased the abundance and species richness of defoliating lepidopterans, as well as it shifted their community composition (Figures 2 and 3, Table 2). We did not observe differences in the species richness, evenness, and composition of the community of lepidopterans among the short‐ and long‐term exclusion when we contrasted them at initial conditions (Appendix S1: Figures S1 and S2, Table S2). Changes in community composition were primarily driven by species nestedness (Appendix S1: Figure S3), where geometrids and noctuids, which generally pupate on the ground (Table 1), were generally favored by livestock exclusion (Figure 4).

**TABLE 2:** The impact of the exclusion of livestock (treatment; areas with livestock, short‐term exclusion, and long‐term exclusion) and presence of livestock (0/1) on lepidopteran abundance, species richness, evenness, and the multivariate variable community composition of the community of defoliating lepidopterans 2 years from the start of the experiment and in the field survey respectively.

Response variable	Predictor	χ^2^/*F*	df	*p*	*R* ^2^
Field experiment					
Abundance	Treatment	11.54	2	**0.003**	0.14
Species richness	Treatment	7.90	2	**0.019**	0.10
Evenness	Treatment	5.23	2	0.073	0.07
Community composition	Treatment	2.80	2, 186	**0.002**	0.03
Field survey					
Abundance	Livestock	22.52	1	**<0.001**	0.12
Species richness	Livestock	60.93	1	**<0.001**	0.23
Evenness	Livestock	3.52	1	0.061	0.03
Community composition	Livestock	8.42	1, 94	**<0.001**	0.08

*Note*: Shown are χ^2^ values (models on abundance, richness, and evenness), *F* values (models on community composition), df, *p* values, and marginal *R* ^2^ values (for full models in models on abundance, richness, and evenness and for each predictor in models on community composition). Significant *p* values appear in boldface.

### The impact of livestock on the community of defoliating lepidopterans

The abundance and species richness of defoliating lepidopterans were highest in long‐term exclusion, but we did not find differences in evenness among treatments (Figure 2, Table 2; Appendix S1: Table S3). Moreover, the community composition of the defoliating lepidopterans changed with the exclusion of livestock (Figure 3, Table 2). Similarly, abundance and species richness in areas without livestock were higher than in areas with livestock in our field survey, but we did not find any impact of the presence of livestock on evenness (Figure 2, Table 1). Furthermore, the presence of livestock also shifted community composition in our field survey (Figure 3, Table 2).

### Vulnerability of lepidopteran species

In the field experiment, the tortricids *A. xylosteana* and *T. viridana* (canopy pupation) were significantly more abundant in areas with livestock and/or in short‐term exclusions than in long‐term exclusions, while the tortricid *Tortricodes alternella* (canopy pupation) was significantly more abundant in the long‐term exclusion than in all the other treatments (Figure 4). Geometrids and the noctuid *Xanthia ruticilla* (ground pupation) were significantly more abundant in long‐term exclusions than in all the other treatments (Figure 4). In the field survey, the noctuid *Colotois pennaria* and the geometrid *Eupithecia irriguata* (ground pupation) were more abundant in areas without livestock than in areas with livestock (Figure 4). Overall, differences in community composition among livestock exclusion treatments were mainly a result of nestedness and only to a minor extent a result of turnover (Appendix S1: Figure S3). Among areas, differences in community composition in areas with and without livestock followed similar patterns than in the field experiment, but the contribution of components was generally more balanced (Appendix S1: Figure S3).

## DISCUSSION

In our study, we evaluated the influence of livestock on the community of defoliating lepidopterans in Mediterranean agroforestry systems, focusing on the effects of intraguild elimination. We employed both experimental and observational methodologies for this purpose. Both our field experiment and survey collectively indicated that the presence of livestock led to a decrease in both the abundance and species richness of defoliating lepidopterans. Moreover, it also resulted in a shift in the composition of their community. The differences in community composition among the exclusion areas were predominantly due to species nestedness. We observed that the extent of livestock exclusion consistently favored species pupating on the ground, especially geometrids and noctuids. Overall, our results underscore that livestock presence alters the composition of defoliating lepidopteran communities in agroforestry systems. This has significant implications for the structure of food webs and the species interactions.

### The impact of livestock on the community of defoliating lepidopterans

Our study revealed that livestock generally exerted a negative influence on the lepidopteran community within Mediterranean agroforestry systems. We observed the highest abundance of lepidopterans and species richness in trees under long‐term exclusion and noted differences in community composition across various treatments. These negative effects on lepidopteran communities could be offset by a positive effect on tree defoliation, but further field studies will be needed to evaluate if livestock can effectively reduce tree defoliation. Our field survey corroborated the findings of our field experiment, indicating that the presence of livestock negatively impacted the lepidopteran community. Given that short‐term exclusions only marginally affected the lepidopteran community, our results suggest that longer periods are necessary to fully observe the impact of livestock on this community. Our findings align with previous studies that evaluated the impact of both wild and domestic large ungulates on various insect guilds, including seed feeders (Bonal et al., 2007; Canelo et al., 2021; Valburg, 1992), aphids (Gish et al., 2011; Losey & Denno, 1998), gallers (Zamora et al., 1993), or ground‐dwelling insects (Suominen et al., 1999). However, the mechanisms underlying intraguild elimination might vary among insect guilds. Intraguild elimination can occur due to livestock's impact on ground through trampling or the direct consumption of plants by grazing or browsing. The susceptibility of the lepidopterans to livestock is determined by the location of their pupation and the duration of this stage, with species pupating in the ground for extended periods—even overwintering—facing the highest predation risk. We identified similarities between defoliating lepidopterans and ground‐dwelling insects (Suominen et al., 1999), as both guilds inhabit the ground and are at risk of being trampled by livestock. Other guilds, such as aphids or gallers, are susceptible to livestock due to the direct consumption of plants (Gish et al., 2011; Losey & Denno, 1998; Zamora et al., 1993). Seed feeders present a double‐risk case, as they are at risk of being trampled when the fruit is on the ground and when the insect burrows into the ground to pupate. They can also be accidentally consumed by livestock, particularly cattle or pigs, within the fruit (Bonal et al., 2007; Canelo et al., 2021; Valburg, 1992). The impact of livestock on insect communities may be more profound than that of wild mammals due to the intensity of livestock management, leading to a simplification of insect communities in these areas. Defoliators and livestock engage in an asymmetric competition for feeding resources, with the balance tipped in favor of defoliators due to their early spring activity. The consumption of tree leaves and flowers by lepidopterans diminishes the fruit availability for livestock (Canelo et al., 2018). However, this impact is later offset when livestock contribute to the reduction of defoliator populations (intraguild elimination).

### Vulnerability of lepidopteran species

Our findings suggest a potential relationship between abundance and changes in community composition. We observed that livestock exclusion consistently favored species that pupate in the ground, while species that pupate on tree branches were significantly more abundant in areas with livestock. This aligns with our expectations, as leaf rollers, which pupate in the canopy, are more protected against livestock, and vulnerable species that pupate on the ground are not compromised in excluded areas. Predominantly, differences in community composition among exclusions were due to species nestedness, with species turnover playing a minor role. Importantly, there were not lepidopteran species inhabiting only areas with livestock (Table 1). This suggests that reducing livestock favors the colonization of previously managed areas by those lepidopteran species that were previously inhabiting these and the surrounding areas. However, the colonization ability of each species is dependent on their mobility and specific life‐history traits (Renault, 2020; Slade et al., 2013; Zheng et al., 2015). For instance, females of many geometrids, such as *Agriopis* spp. or *Erannis defoliaria*, are wingless, which reduces their chances of colonizing areas that had livestock (Renault, 2020). Other species could limit their movement when they are attracted by pheromones associated with reproduction (Bengtsson et al., 2014). Conversely, species as *L. dispar* or *T. viridana*, which historically caused intense herbivore damage in the Iberian Peninsula (Bernal et al., 2023; Soria & Notario, 1990), were unaffected by the presence of livestock. This relationship raises an intriguing ecological question: Does the presence of livestock increase the chances of pest outbreaks of favored species? Further studies are needed to evaluate which species traits are most crucial for insect colonization of managed agroforestry systems, and how the presence of livestock might affect herbivory rates in trees.

## CONCLUSIONS

In our study, we found that livestock generally has a detrimental effect on the lepidopteran community within agroforestry systems. These detrimental effects appear to be stronger than other potential indirect beneficial effects of livestock, such as the nitrogen addition to trees from manure (Piñeiro et al., 2006), which can enhance leaf quality and would favor defoliating lepidopterans. It is plausible that species which are especially susceptible to livestock may require several growth cycles to colonize trees in areas previously subjected to management. Our findings offer insights into which locations are more likely to harbor lepidopteran species that could emerge as a pest, a matter of significant economic and ecological concern in agroforestry systems. This research underscores agroforestry systems as nature‐based solutions for controlling tree pests while preserving biodiversity.

## AUTHOR CONTRIBUTIONS

Álvaro Gaytán analyzed the data and wrote the manuscript. Raúl Bonal and Tara Canelo conceived the idea and supervised the study. Álvaro Gaytán, Tara Canelo, Carlos Pérez‐Izquierdo, and Raúl Bonal carried out the fieldwork and revised the latest version of the manuscript.

## CONFLICT OF INTEREST STATEMENT

The authors declare no conflicts of interest.

## Supporting information


Appendix S1.

## Data Availability

Data and code (Gaytán et al., 2024) are available in Figshare: https://doi.org/10.6084/m9.figshare.27868575.
