# Supplementary material for: Livestock shifts lepidopteran herbivore community due to intraguild elimination in Mediterranean agroforestry systems
Source: Ecol Appl. 2025 Jan 11;35(1):e3088. doi: 10.1002/eap.3088 (PMC11724503; doi:10.1002/eap.3088)
Supplement: Supplementary file 1 — Appendix S1. [file EAP-35-e3088-s001.pdf]

Supporting Information. Gaytán, M., Canelo, T., Pérez-Izquierdo, C., Bonal, R. Livestock shifts lepidopteran herbivore community due to intraguild elimination in Mediterranean agroforestry systems. Ecological Applications.

APPENDIX S1: Livestock density in the study areas. Status of the community of defoliating lepidopterans at the starf of the experiment. Partitioning of the total β-diversity in the community of defoliating lepidopterans among treatments. Post-hoc analyses comparing abundance, species richness and evenness on early and late spring in each treatment.

Table S1. Number of animals and livestock units (LSU, according to the pertinent legislation) per hectare at each study areas (experiment and survey) and the livestock present in each area (pigs, cattle or sheep).

Area	Livestock	Type	Animals/ha	L.S.U./ha
Guijo de Granadilla 1	Pigs	Experiment	1.00	0.14
Guijo de Granadilla 2	Cattle	Experiment	1.00	0.60
Malpartida de Plasencia	Sheep	Experiment	2.10	0.32
Arroyo de la Luz	Cattle	Survey	2.50	1.50
Bohonal de Ibor	No livestock	Survey	0.00	0.00
Piedrahita 1	Cattle	Survey	2.00	1.20
Piedrahita 2	No livestock	Survey	0.00	0.00
Puebla de Alcocer	Sheep	Survey	3.50	0.53
Rosalejo	Sheep	Survey	4.00	0.60
Zarza Capilla	No livestock	Survey	0.00	0.00

Table S2. The impact of the exclusion of livestock (Treatment; sites with livestock, short-term exclusion and long-term exclusion) on species richness, evenness and community composition of the community of defoliating lepidopterans at the start of the experiment. Shown are χ^2 values (models on abundance, richness and evenness), F-values (models on community composition), degrees of freedom, P-values and marginal R^2 values (for full models in models on abundance, richness and evenness and for each predictor in models on community composition). Significant P-values are shown in bold.

Response variable	Predictor	χ^2/F	df	P-value	R^2
**Field experiment**					
Abundance	Treatment	8.23	2	**0.016**	0.07
Species richness	Treatment	8.20	2	**0.017**	0.07
Evenness	Treatment	0.03	2	0.986	<0.01
Community composition	Treatment	1.55	2, 109	0.102	0.03

Table S3. Results of post-hoc analyses comparing abundance, species richness and evenness on early and late spring in each treatment (control, short-term exclusion and long-term exclusion). Shown are the groups compared, estimates, standard errors (SE), t-ratios and P-values. Significant P values (P < 0.05) are shown in bold.

Response variable	Contrast	Estimate	SE	t-ratio	P-value
Abundance	Areas with livestock vs. Short term exclusion	1.95	3.54	0.55	0.847
Abundance	Areas with livestock vs. Long term exclusion	11.40	3.58	3.19	**0.006**
Abundance	Short term exclusion vs. Long term exclusion	9.46	3.59	2.64	**0.028**
Species richness	Areas with livestock vs. Short term exclusion	0.88	0.81	1.11	0.513
Species richness	Areas with livestock vs. Long term exclusion	2.23	0.80	2.76	**0.020**
Species richness	Short term exclusion vs. Long term exclusion	1.35	0.81	1.66	0.227
Evenness	Areas with livestock vs. Short term exclusion	0.02	0.02	0.84	0.680
Evenness	Areas with livestock vs. Long term exclusion	0.05	0.02	2.24	0.072
Evenness	Short term exclusion vs. Long term exclusion	0.03	0.02	1.40	0.345

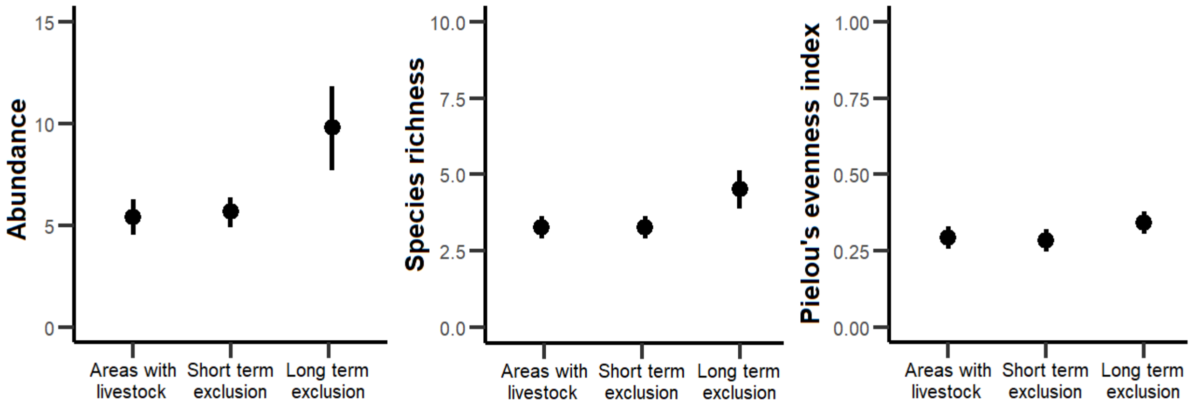

Figure S1. The impact of treatments (sites with livestock, short-term exclusion and long-term exclusion; left panels) on abundance, species richness and evenness at the start of the experiment. Circles represent means and bars represent standard errors.

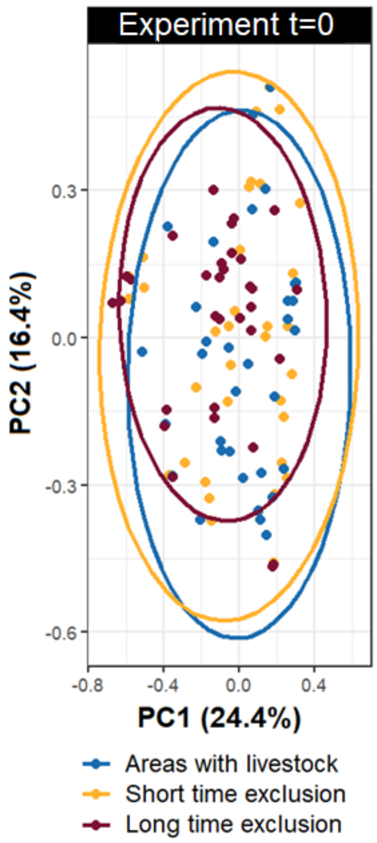

Figure S2. Differences in the community composition of defoliating lepidopterans among treatments (sites with livestock, short-term exclusion and long-term exclusion) at the start of the experiment. Visualization is based on principal component analyses (PCA) using Bray–Curtis metrics.

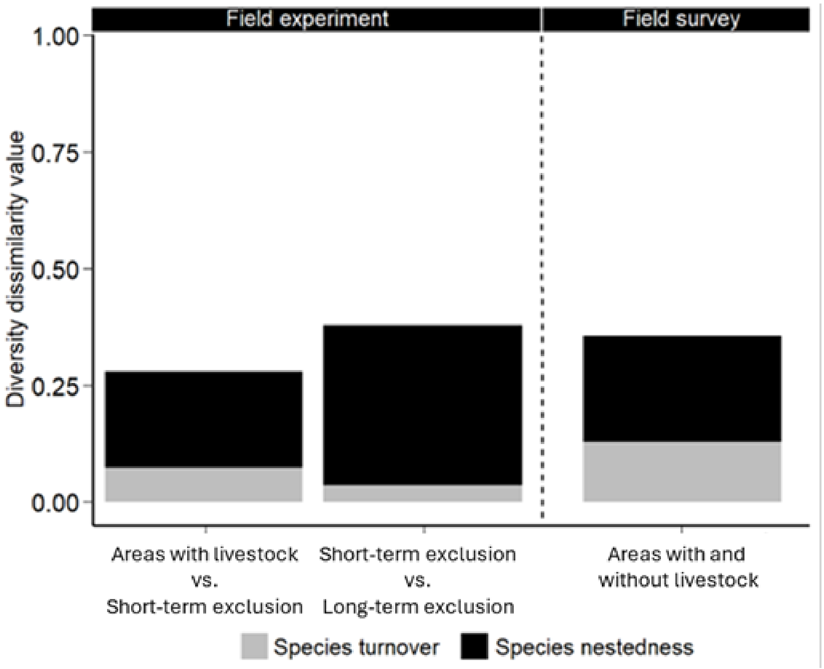

Figure S3. Partitioning of the total β-diversity in the community of defoliating lepidopterans among treatments (field experiment; areas with livestock vs. short-term exclusion and short-term exclusion vs. long-term exclusion) and between areas with and without livestock (field survey) into the components of species turnover (grey) and nestedness (black) in pairwise comparisons.
